# Study on simulation effect of physical and chemical characteristics of sausage by sausage model system

**DOI:** 10.3389/fnut.2024.1408618

**Published:** 2024-05-22

**Authors:** Lili Ji, Chunyan Zhou, Yanan Zhou, Qing Nie, Yi Luo, Rui Yang, Shu Wang, Jiawen Ning, Jiamin Zhang, Ying Zhang

**Affiliations:** ^1^Meat Processing Key Laboratory of Sichuan Province, Chengdu University, Chengdu, Sichuan, China; ^2^Chengdu Huixin Foods Co., Chengdu, China

**Keywords:** *Staphylococcus xylosus*, sausage, physicochemical properties, flavor characteristics, fermentation model

## Abstract

**Introduction:**

The incorporation of *Staphylococcus xylosus* in sausage production is hypothesized to affect various physicochemical properties and flavor profiles of sausages. This study aimed to evaluate the simulation of these features in a sausage model and establish its applicability for in vitro studies.

**Methods:**

Both a control and an experimental model, inclusive of *Staphylococcus xylosus*, were assessed for changes in physicochemical indexes (pH and water activity, Aw) and the concentration of flavoring components (esters and aldehydes). Thiobarbituric acid reactive substances (TBARS) values were also measured to evaluate lipid oxidation.

**Results:**

The introduction of *Staphylococcus xylosus* resulted in no significant changes in pH and Aw between the sausage and the model. However, there was a considerable increase in the content of volatile flavor compounds, specifically esters and aldehydes, in the experimental groups compared to the control. Additionally, the TBARS values in experimental groups were significantly lower than those in the control group at the end of the testing period.

**Discussion:**

The findings indicate that *Staphylococcus xylosus* plays a critical role in enhancing the flavor profile of sausages through the increased synthesis of volatile compounds and inhibiting fat oxidation. The sausage model effectively simulated the physicochemical and flavor index responses, demonstrating its potential utility for further *in vitro* research on sausage fermentation and preservation techniques.

## 1 Introduction

China has long enjoyed sausage as a traditional pork product because of its distinct flavor and texture. The flavor, color, and texture of sausage are all strongly correlated with the microflora. Industrialized sausage production uses an increasing number of fermentation microorganisms to control the quality and stability of the sausages. Examining how various microorganisms affect the safety, flavor, and quality of sausage has gained popularity as a research area. *Staphylococcus* spp. are dominant microorganisms in the maturation period of traditional Chinese cured meat products, such as ham, sausage, and bacon. It has been discovered that *Staphylococcus* spp. can improve the color and texture, inhibit fat oxidation, delay spoiling, and promote the decomposition of proteins and fats to enhance the product flavor (1–3). It has been shown that the addition of *Staphylococcus xylosus* significantly enhances the protein hydrolysis of sausage, hydrolyzing proteins into small peptides as well as a large number of flavor compounds, which improves the flavor of sausage (4). Protease with strong protein hydrolysis activity is secreted by *Staphylococcus xylosus*, which can also improve the flavor and quality of sausage (5). Furthermore, *Staphylococcus xylosus* and lactic acid bacteria in combination can enhance the enrichment of free amino acids in meat products that have undergone fermentation (6). The study of *Staphylococcus xylosus* fermenters has grown in depth as beneficial staphylococci are employed in the manufacturing of meat products more and more.

Most of the studies were conducted by directly adding the target microorganisms to the sausages and determining their effects by measuring various indicators, which can be affected by the microorganisms in the raw materials and the environment, the climatic conditions of the production, etc. Moreover, the process of making sausages is complicated and time-consuming, which is especially unfavorable for the prediction and mechanistic study of microbial effects. Therefore, some modeling systems have been developed to conduct mechanistic studies. Effects of nitrate and staphylococci on the color of cooked cured meat products using a dry sausage model by Waga et al. (7) and Huang et al. (8). Chaves-López et al. (9) and Mi et al. (10) investigated *Saccharomyces cerevisiae*’s capacity for protein hydrolysis using a fermentation modeling system. Tjener et al. (11) created a modeling system for fermented meat to study the impact of microbes on the generation of volatiles. To examine the impact of *Penicillium nalgiovense*, *Pediococcus pentosaceus*, sodium ascorbate, sodium nitrate, and temperature on the 79 volatiles produced during incubation, Sunesen et al. (12) created 32 agar sausage models. Among these, it was discovered that a modeling system could assess how well seven strains of *Debaryomyces hansenii* produced fragrance chemicals (13). De Almeida conducted a thorough analysis of this model and discovered that it could replicate the responses of sausages throughout their manufacturing (14). The model was also applied by Zhao et al. (15) to screen a strain of *Lactobacillus* that could significantly enhance the quality of sausage. Consequently, in this study, a sausage model was used to compare with sausage, and *Staphylococcus xylosus* was chosen to be added to the sausage fermentation model system mentioned above. This allowed for a comparison and analysis physicochemical indexes and microbiological properties of the products, as well as a gas chromatography–mass spectrometry (GC–MS) analysis of the volatile compound composition. Additionally, the study examined the effect of *Staphylococcus xylosus* on the flavor quality of sausage *in vitro* and *in vivo*, as well as the simulation of the sausage model on the physicochemical properties of sausage and to further validate the stability of the model system. The aim of this study is to provide a stable model to reduce the interference of raw materials, external environment and other factors on the results of the study; to provide a new model system for the mechanistic study of the flavor of fermented meat products.

## 2 Materials and methods

### 2.1 Materials

*Staphylococcus xylosus*, CS300, Cohansen (China) Co., 10 g/bag. Pork, purchased from Shiling Town Farmers’ Market, fresh pork slaughtered on the same day. Thiobarbituric acid, Chengdu Kolon Chemical Co., Ltd.; trichloroacetic acid, Chengdu Kolon Chemical Co., Ltd.; 2,4,6-trimethylpyridine standard, Sigma Corporation, USA; mannitol medium, Hangzhou Best Biotechnology Co.

### 2.2 Production of sausage model

Referring to the method of Liliana (13), with slight modifications, for modeling.

Protein extraction: fresh fat-free and connective tissue-free dorsal long muscle mince was used for preparation. Add 0.1 mol/L Tris–HCl and 20 mmol/L EDTA, and homogenize the meat with a blender at pH 7.0 (1,4 w/v). The meat was centrifuged at 10,000 r/min for 15 min at 4°C and the supernatant was discarded. This procedure was repeated 3 times to remove the supernatant containing myoplasmic proteins, and the pellet containing myofibrillar proteins was stored at −20°C until use.

Buffer preparation (g/L): NaCl (30), glucose (10), sodium nitrite (0.15) and a solution similar to the amino acid content in fresh pork were prepared by mixing and sterilized by filtration with a sterile 0.22 μm filter membrane.

Modeling: The sausage model system was prepared using extracted myogenic fibrin, fat and buffer solution. Each 500 mL of prepared buffer solution was mixed with 175 g of myogenic fibrin and 75 g of surimi-like pig back fat in a blender (8,000 r/min, 0.5 min, repeated 3 times). *Staphylococcus xylosus* was inoculated at 5 lg CFU/g, and the same volume of saline was added to the blank group; finally, 2 mL of 56 mmol/L gluconolactone was added to gel the model. The uninoculated strain sausage model was labeled as CT, and the inoculated sausage model was labeled as SX. The CT and SX groups were placed in constant temperature incubation at 12°C, and the samples were taken and analyzed on the 0th and 14th days of production.

### 2.3 Sausage preparation

Lean meat: fat (7:3) with 2.5% salt, 5 lg CFU/g inoculated with *Staphylococcus xylosus*, labeled SXS; and sausages without *Staphylococcus xylosus* as a control, labeled CTS. All sausages were hung at 8–12°C for 7 d, stored under vacuum at room temperature, and sampled and analyzed on the 0th and 30th day of preparation.

### 2.4 Indicator measurement

#### 2.4.1 pH

The pH meter was inserted in the model for determination, and the lean part of the sausage was selected for determination, and each sample was measured three times in parallel and the average value was taken.

#### 2.4.2 Aw

Take about 2 g of the homogenized sample, spread it over the sample measuring dish and measure it in the moisture activity meter, wait for the instrument reading to stabilize and read the Aw value.

#### 2.4.3 TBARS

5 g ground sample was mixed with 50 mL 0.75 g/L trichloroacetic acid solution (containing 0.01 g/L ethylenediamine tetraacetic acid), shaken well and sealed with a plug. After oscillating at 50°C for 30 min, it was cooled to room temperature. Filter with double-layer quantitative slow speed filter paper, discard the primary filtrate, and collect the remaining filtrate for later use. 5 mL of the filtrate was mixed with 5 mL of 0.02 mol/L thiobarbituric acid solution and then heated in a water bath at 90°C for 30 min. Trichloroacetic acid solution with the same concentration was used as blank control. The absorbance value was measured at 532 nm, and the result was expressed as the content of malondialdehyde (mg/kg sample). The determination was run in triplicate.

#### 2.4.4 Number of cocci

Take 25 g homogenized sample in a sterile homogenization bag, add 225 mL sterile saline, beat in homogenizer for 5 min, inoculate the appropriate concentration in mannitol medium, incubate at 30°C for 48 h, count the plates with 30–300 colonies, and calculate the number of cocci.

#### 2.4.5 Total sulfhydryl content

Myofibrillar protein extraction referred to the method of Park et al. (16), followed by the determination of total sulfhydryl content, the specific steps are: weigh 0.1 g of tissue samples, add 1 mL of the extraction solution, ice bath homogenization, centrifugation at room temperature (8,000 g, 10 min), and take the supernatant to be measured; enzyme labeling instrument preheating for more than 30 min, and absorbance was measured at the wavelength of 412 nm.

#### 2.4.6 Surface hydrophobicity (H_0_)

The H_0_ was characterized by the amount of myofibrillar protein binding to bromophenol blue, and the assay was referred to the method of Chelh et al. (17), myofibrillar protein was adjusted to a concentration of 5 mg/mL with Na_2_HPO_4_-NaH_2_PO_4_ buffer solution (0.1 mmol/L, pH 7.5). 1 mL of the above solution and 200 μL of 1 mg/mL bromophenol blue solution were taken and mixed. The solution was mixed, vortexed and shaken for 10 min and then centrifuged (5,000 r/min, 4°C, 15 min); the supernatant was taken and the absorbance value was measured at 595 nm. The blank control was replaced by 1 mL phosphate buffer, and each sample was measured three times in parallel. The formula was as follows:


$$\mathrm{Surface}\;\mathrm{hydrophobicity}/\mathrm{\mu g}=200\times \frac{A_{0}-A}{A_{0}}$$

200 is the mass of bromophenol blue, μg; A_0_ is the absorbance value of the blank control group; A is the absorbance value of the sample group.

#### 2.4.7 Volatile flavor substances

The determination of volatile flavor compounds of sausage samples with reference to the method of Zhao et al. (18). The sample was weighed and sealed in a 15 mL headspace vial, and the parameters of the CTC autosampler were set as follows: heating box temperature 75°C, heating for 45 min, sample extraction for 20 min, and resolving for 5 min; in the gas chromatography without shunt mode, the temperature of the inlet port was set at 250°C, and the carrier gas was helium with a flow rate of 1.0 mL/min under a pressure of 32.0 kPa; the HP-5MS UI column (30 m × 0.25 mm, 0.25 μm) was used. 5MS UI column (30 m × 0.25 mm, 0.25 μm); the temperature increase program was set as follows: the starting temperature was 40°C, held for 1 min, then increased to 85°C at 3°C/min, held for 3 min, then increased to 105°C at 3°C/min, held for 2 min, then increased to 165°C at 12°C/min, held for 1 min, then increased to 230°C at 10°C/min, held for 1 min. 230°C, held for 1 min. Electron energy 70 eV; ion source temperature 230°C, quadrupole temperature 150°C; detector voltage 350 V; mass scan range (m/z): 40–500. Compound data were identified in the NIST 14 L mass spectrometry database with a mass match of >80%.

### 2.5 Data analysis

All test items were repeated three times and expressed as mean ± relative deviation, using SPSS 25 software to perform significance analysis. GraphPad Prism 9.0.0 plotted the variation in physicochemical properties of sausages. Supervised orthogonal partial least squares models (OPLS-DA) were plotted by SIMCA (14.1) and screened for characteristic flavor substances with significant projected variable values and Student’s test (VIP > 1, *p* < 0.05).

## 3 Results

### 3.1 pH

**Figure 1:**
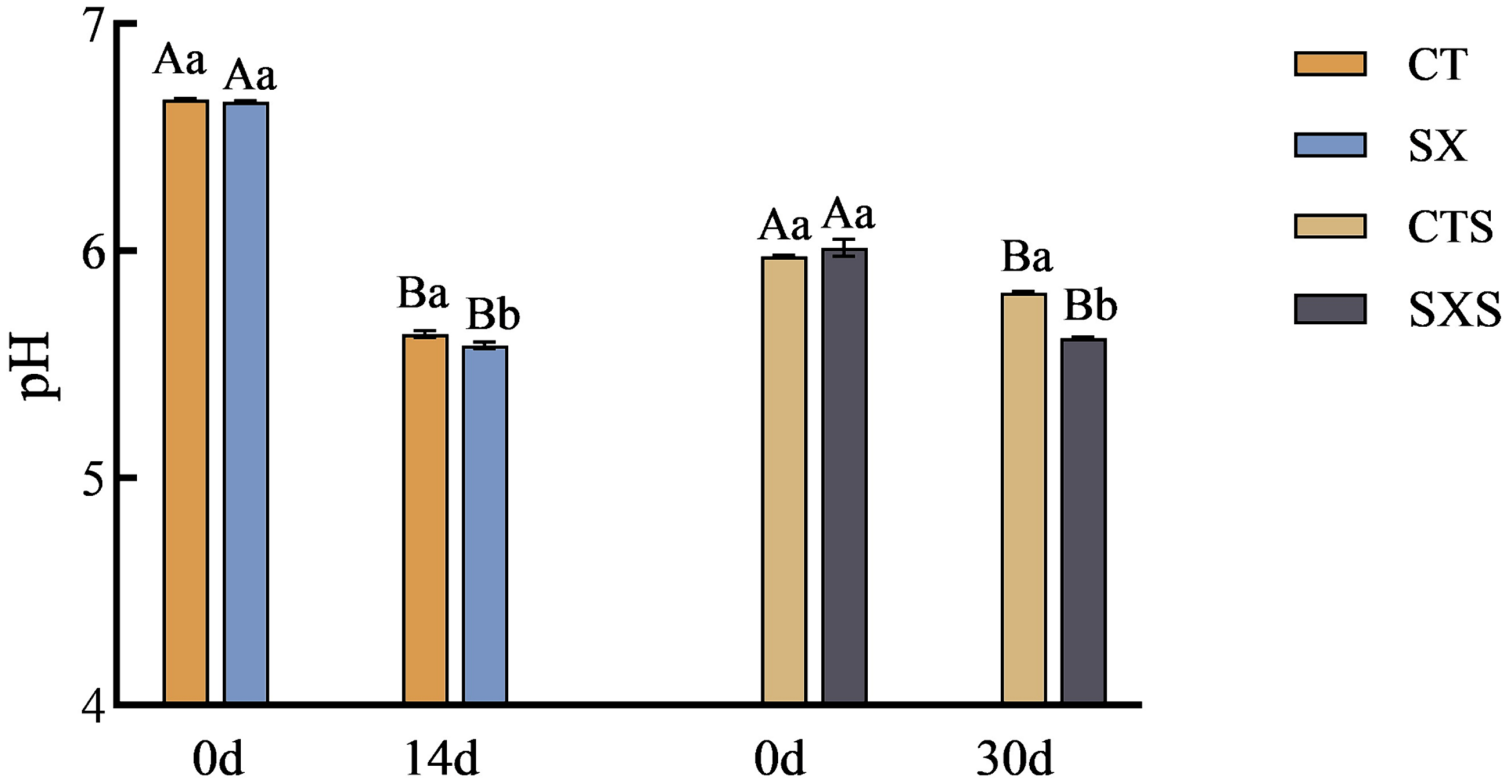
pH changes in models and sausages. CT, Sausage model control group; SX, Sausage model experimental group; CTS, Sausage control group; SXS, Sausage experimental group. Different capital letters in the graph indicate significant differences between time points within groups (*p* < 0.05), and different lowercase letters indicate significant differences between different treatment groups at the same time point (*p* < 0.05), as follows.

The pH values in the model and sausage are shown in Figure 1. The pH values of the model and sausage started to decrease from the time of preparation, and the decrease was more pronounced in the model than in the sausage. The pH at the end of fermentation in the sausage model decreased to 5.58 in the SX group and 5.63 in the CT group; the pH of the SX group was significantly lower than that of the CT group (*p* < 0.05). At the end of sausage fermentation, the pH was 5.62 in the SXS group and 5.82 in the CTS group; similarly, the pH of the SXS group was significantly lower than that of the CTS group (*p* < 0.05). The growth of lactic acid bacteria was highly correlated with pH changes. The large number of lactic acid bacteria in the early stages of fermentation may have accelerated the breakdown of carbohydrates into small molecule compounds like lactic acid and acetic acid, as well as the large amount of organic acid accumulation that also contributed to the system’s rapid pH decline in the treatment groups (19, 20). *Staphylococcus xylosus* was able to lower the pH of sausage in this experiment, as demonstrated by the *in vivo* and *ex vivo* results. This suggests that the sausage model employed in the study was able to accurately replicate the pH trend of sausage.

### 3.2 Aw

**Figure 2:**
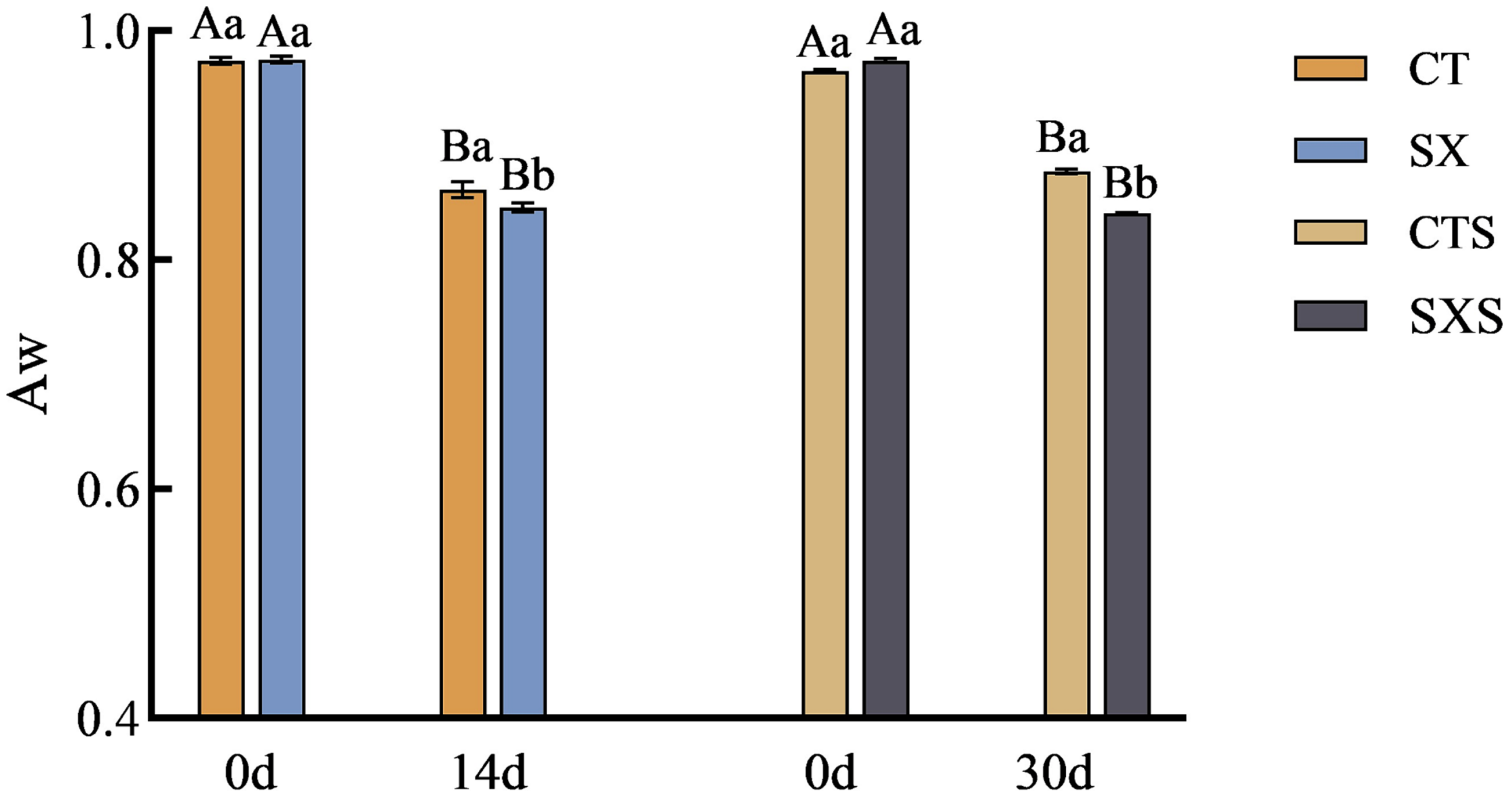
Aw changes in models and sausages.

In addition to properly reflecting the physical and chemical characteristics of the water in food, the moisture activity value can also be used to predict the growth of microorganisms in food by indicating the extent to which the bacteria are utilizing the water. Reduced moisture activity not only helps to extend the food’s shelf life and block chemical changes, but it also works to stop germs from growing and reproducing, maintaining food safety and quality. The model and the sausage *in vitro* both showed a declining trend in Aw, as shown in Figure 2; at the end of fermentation, the SX and SXS groups had considerably lower Aw values than the control group (*p* < 0.05). Following a 14-day fermentation period, the Aw value dropped in the SX group from 0.975 to 0.846 and in the CT group from 0.971 to 0.861. Following 30 days of sausage fermentation, the Aw dropped in the SXS group from 0.974 to 0.841 and in the CTS group from 0.965 to 0.877. The Aw values of sausages in general fell between 0.987 to 0.795, suggesting that the model did a better job of simulating fluctuations in moisture activity in sausages. The proliferation of microorganisms in the sample is correlated with changes in moisture activity; the more bacteria multiply, the more Aw is consumed. It is evident that the Aw value of sausage can be reduced more quickly when *Staphylococcus xylosus* is added. It has been demonstrated that Aw and lower pH can prevent the growth of dangerous germs (21). This demonstrates how adding *Staphylococcus xylosus* to sausage can improve its safety and quality. This result is consistent with the findings of Lorenzo et al. (22).

### 3.3 TBARS

**Figure 3:**
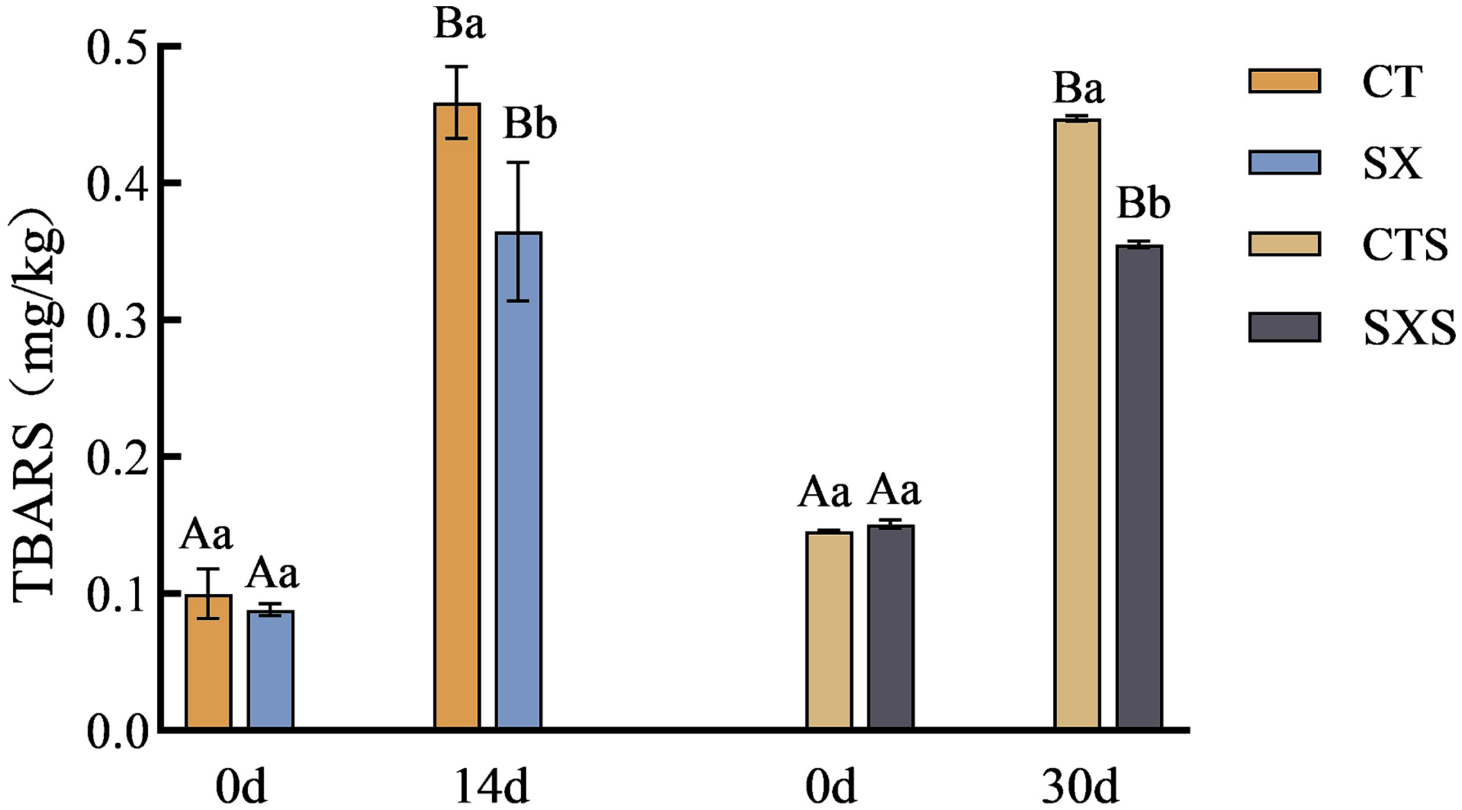
TBARS changes in models and sausages.

Lipid oxidation is related to the flavor of the sausage, and TBARS measures the amount of oxidation by quantifying the secondary products that result from oxidation (23). The lipids continued to oxidize during the fermentation of the samples in each group, as shown in Figure 3, and the degree of secondary oxidation rapidly deepened (24). The TBARS levels in the model and the sausages also grew gradually with the production time. However, samples spiked with *Staphylococcus xylosus* had lower TBARS levels compared to controls. It has been shown that *Staphylococcus xylosus* protease inhibits lipid oxidation in Harbin dry sausage (5). In the *in vitro* model, the TBARS content of the SX group at day 14 was 0.37 mg/kg, which was significantly lower than that of the CT group at 0.46 mg/kg (*p* < 0.05). At 30 days, the SXS had TBARS values of 0.36 mg/kg and the control group had 0.45 mg/kg. *Staphylococcus xylosus* was discovered to have an inhibitory impact against lipid oxidation both *in vivo* and *in vitro* based on test findings. This could be because, as it multiplies, *Staphylococcus xylosus* secretes enzymes that have antioxidant properties, lowering the concentration of TBARS.

### 3.4 Coccus count

**Figure 4:**
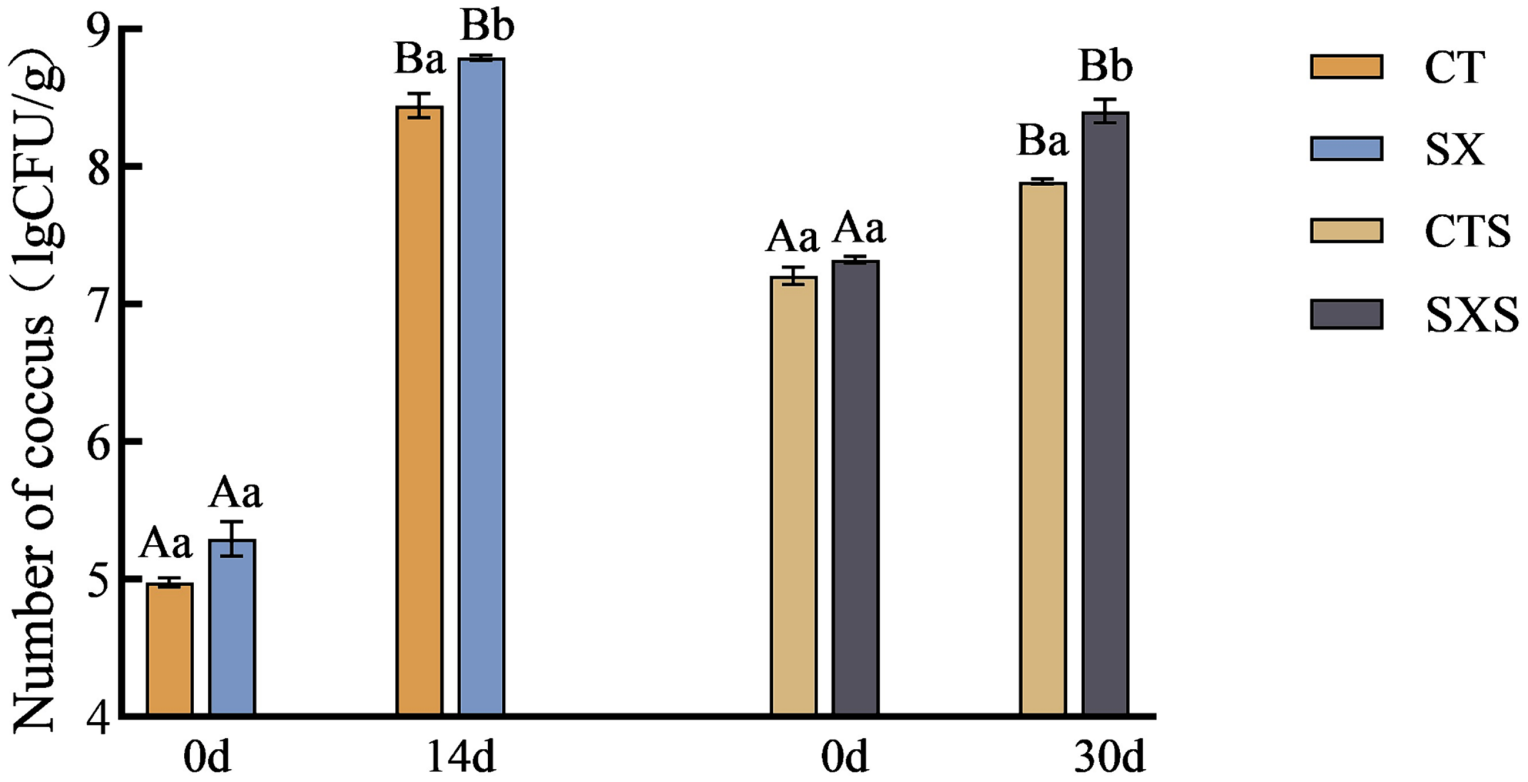
Number of coccus in models and sausages.

Figure 4 illustrates how the number of cocci grew progressively in the sausage and model. 5.29 lg CFU/g in the SX group at 0 d and 8.79 lg CFU/g at 14 d. Although there were initially more cocci in the sausage than there were in the model, the growth rate was slower. From 7.32 lg CFU/g to 8.40 lg CFU/g, the SXS increased. While the initial cocci counts in the sausage model were much lower than in the sausage, on day 14 they were significantly greater than in the sausage on day 30, indicating that the environmental circumstances in the sausage model were more stable and the culture conditions more conducive to cocci proliferation.

### 3.5 Total sulfhydryl content

**Figure 5:**
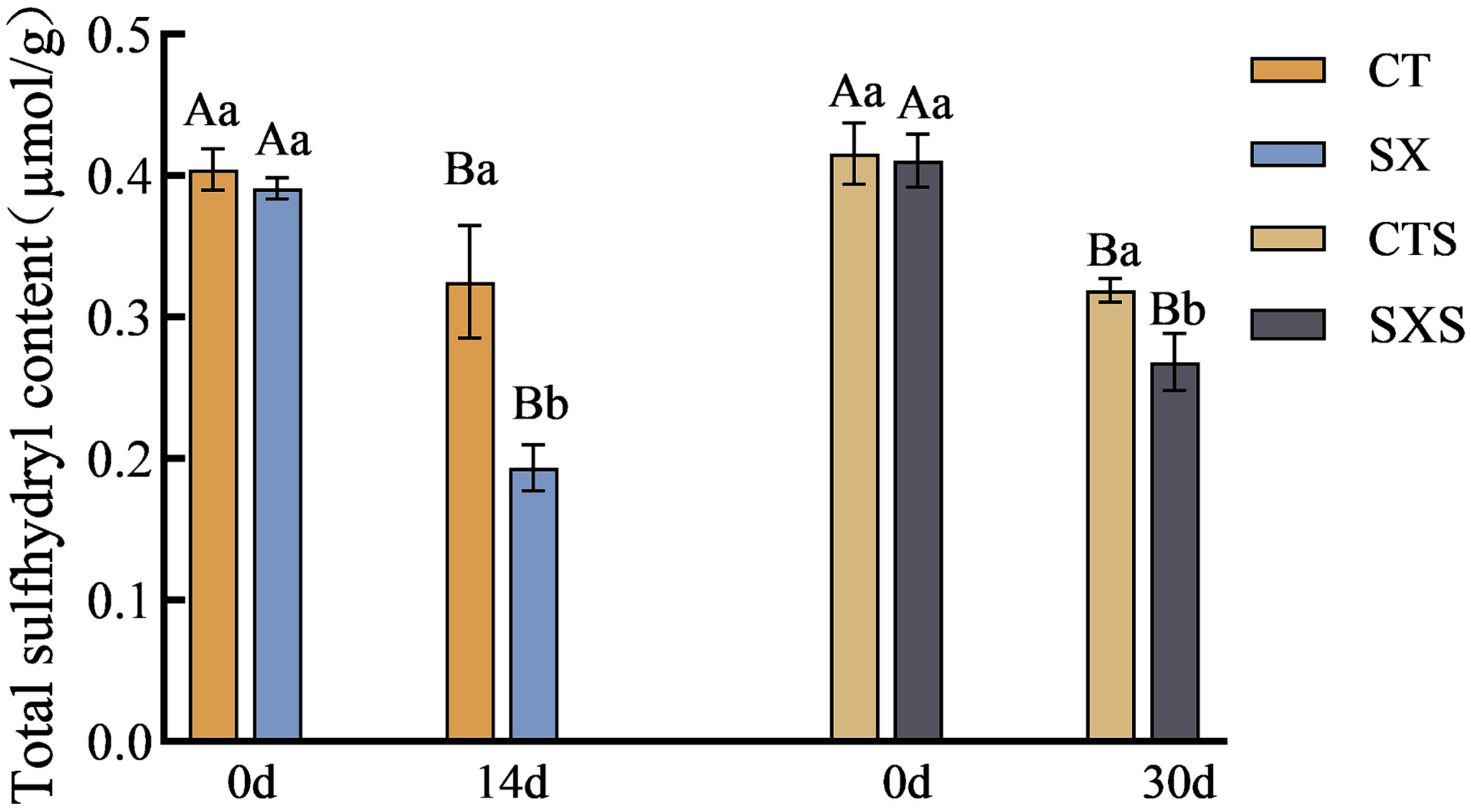
Total sulfhydryl content in models and sausages.

Sulfhydryl concentration is progressively decreased throughout protein denaturation and breakdown; it is intimately linked to protein structure and function and can be used to infer the oxidation state of proteins. The total sulfhydryl concentration variations in the sausage model and sausage at different times are depicted in Figure 5. On the fourteenth day of the sausage model, CT had a total sulfhydryl level of 0.32 μmol/g, whereas SX, which had *Staphylococcus xylosus* added, had a total sulfhydryl content of 0.19 μmol/g. At the 30-day mark in the sausage group, the total sulfhydryl content of CTS and SXS was 0.32 μmol/g and 0.27 μmol/g, respectively. The findings of the experiment demonstrated that, at the conclusion of processing, the total sulfhydryl concentration of the experimental group that had *Staphylococcus xylosus* added was significantly lower than that of each control group (*p* < 0.05). The protease generated by *Staphylococcus xylosus* may encourage protein hydrolysis and disrupt their spatial structure, which could lead to the oxidation of sulfhydryl groups into sulfonic, sulfinic, and sulfinic acids or the conversion of sulfhydryl groups to disulfide bonds, lowering the overall sulfhydryl content (25).

### 3.6 Surface hydrophobicity

**Figure 6:**
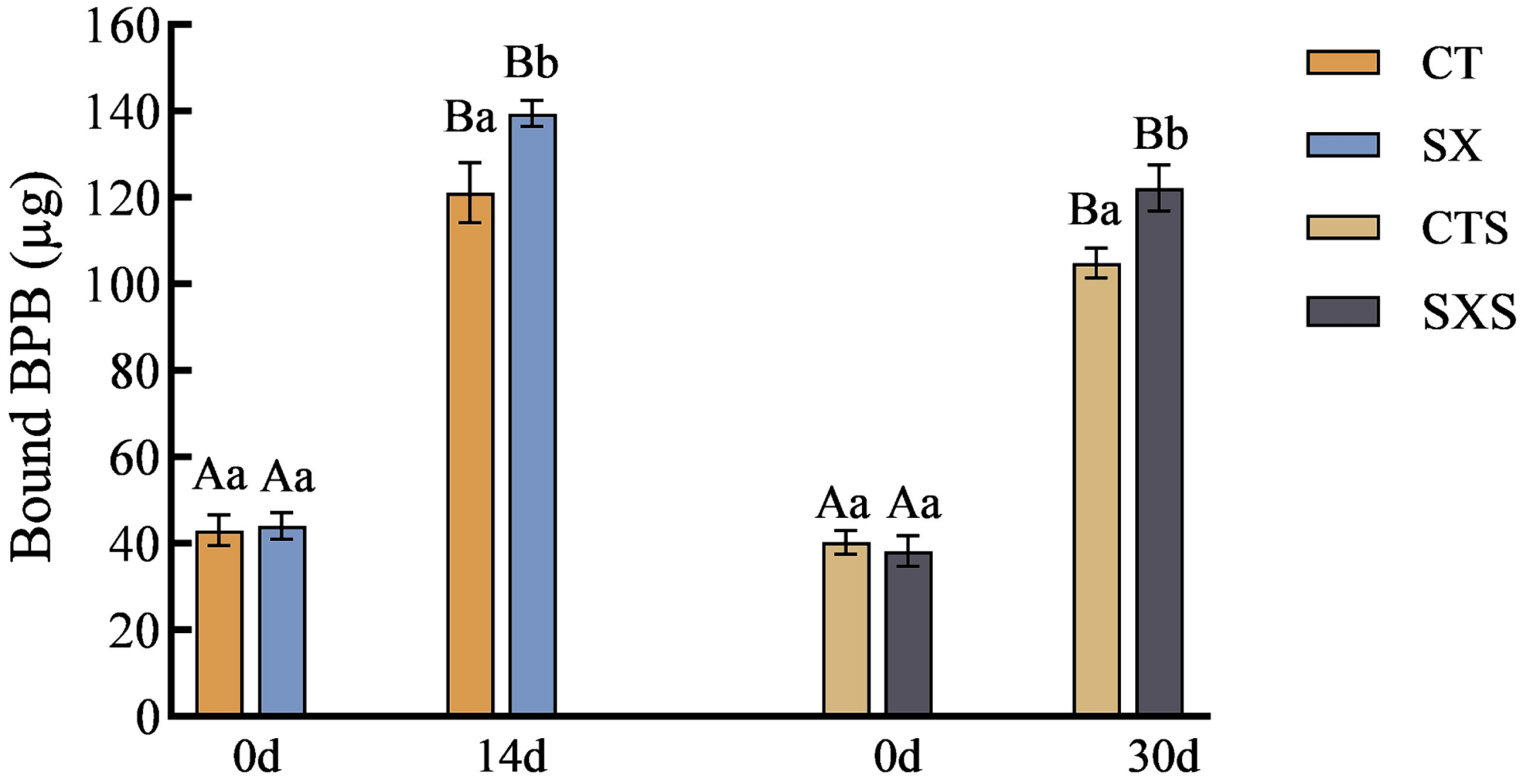
Surface hydrophobicity changes in models and sausages.

The conformation and functional characteristics of a protein molecule are intimately linked to its H_0_. The H_0_ value can objectively reflect the number and distribution of hydrophobic groups on the surface of proteins, which has become one of the effective parameters for assessing the hydrophobicity of proteins (26). Generally, the amount of hydrophobic amino acids bound to bromophenol blue in proteins is used to indicate the size of the H_0_ (27). The variations in the surface hydrophobicity of myofibrillar proteins in the various treatment groups of sausage and the sausage model are displayed in Figure 6. The H_0_ of SX reached 139.45 μg by day 14 in the sausage model group, which was significantly higher than that of 121.14 μg in the CT group (*p* < 0.05). The H_0_ of SXS by day 30 in the sausage group was 122.23 μg, which was significantly higher than that of 104.84 μg in the CTS (*p* < 0.05). Research has demonstrated that the exposure of protein hydrophobic groups can lead to an increase in the quantity of flavor compound binding sites, thereby fostering the production of taste compounds (28). The addition of *Staphylococcus xylosus* increased the H_0_ of both the sausage and the sausage model, indicating that the addition of *Staphylococcus xylosus* enhances the flavor of sausage by promoting the exposure of hydrophobic protein groups.

### 3.7 Changes in volatile flavor substances

Solid-phase microextraction in conjunction with GC–MS was utilized to assess and determine the composition of volatile flavor compounds and their absolute contents of the model samples on day 14 and sausage samples on day 30 in order to look into the impact of *Staphylococcus xylosus* on the flavor profile of sausages (Table 1). It was discovered that following fermentation, the taste component levels of SX and SXS both significantly increased. A total substance content of 17909.69 μg/kg was found for 12 volatile compounds in the CT group, whereas a substantially greater total substance content of 21104.97 μg/kg was found for 17 volatile compounds in the SX group. The CTS group had a total of 22 volatile compounds detected, with a substance concentration of 1417.14 μg/kg, whereas the SXS group had 26 volatile compounds discovered, with a substance content of 2913.54 μg/kg. Alcohols, ketones, aldehydes, and esters constituted the majority of the volatile taste components in the SX and SXS groups, according to a classification of the volatile chemicals found there. Furthermore, a minor quantity of acids was found in the SXS group.

**Table 1:** Variation of absolute content of volatile compounds (μg/kg) in the model and sausages.

Serial number	Compound	Model 14d		Sausage 30d	
		CT	SX	CTS	SXS
	Alcohols				
1	3-methyl-1-Butanol	3095.72 ± 262.07	2766.79 ± 175.98	28.36 ± 28.44	38.17 ± 33.15
2	2-methyl-1-Propanol	488.73 ± 28.23	347.20 ± 37.05	n.d	n.d
3	Isopropyl Alcohol	1191.84 ± 112.24	n.d	n.d	n.d
4	[R-(R*,R*)]-2,3-Butanediol	3351.91 ± 220.04	2438.81 ± 336.60	n.d	49.17 ± 14.57
5	1-Hexanol	n.d	66.59 ± 11.27	n.d	161.20 ± 22.55
6	6-Methoxy-2-hexanol	n.d	n.d	120.21 ± 19.06	n.d
7	1,2:5,6-Dianhydrogalactitol	n.d	n.d	32.09 ± 7.32	n.d
8	1-Octen-3-ol	n.d	n.d	17.28 ± 8.74	51.86 ± 5.62
9	Linalool	n.d	n.d	8.10 ± 2.94	n.d
10	Phenylethyl Alcohol	n.d	121.34 ± 6.74	58.96 ± 0.59	115.21 ± 1.82
11	2,3-Butanediol	3482.47 ± 329.91	4397.31 ± 99.53	n.d	n.d
12	alpha.-methyl-Benzenemethanol	67.29 ± 81.10	n.d	n.d	n.d
	Esters				
13	Decanoic acid, ethyl ester	24.15 ± 1.12	220.96 ± 1.08	n.d	n.d
14	3-propoxy-1-Propene	n.d	420.85 ± 33.15	n.d	n.d
15	4-methyl-2-Oxetanone	n.d	444.23 ± 22.81	n.d	n.d
16	Acetic acid ethenyl ester	163.19 ± 41.26	n.d	n.d	n.d
17	Butanoic acid, 2-methyl-, ethyl ester	n.d	n.d	2.84 ± 2.52	n.d
18	Butanoic acid, 3-methyl-, ethyl ester	n.d	n.d	17.07 ± 9.01	2.97 ± 5.15
19	Formic acid, hexyl ester	n.d	n.d	126.10 ± 58.22	n,d
20	n-Caproic acid vinyl ester	n.d	n.d	n.d	36.43 ± 0.91
	Acids				
21	3-methyl-Butanoic acid	n.d	n.d	28.46 ± 5.64	252.25 ± 5.99
22	Acetic acid	n.d	n.d	n.d	58.48 ± 19.68
	Ketones				
23	Acetoin	5272.91 ± 450.77	7920.04 ± 1780.83	164.27 ± 31.39	583.31 ± 52.96
24	2-Heptanone	n.d	n.d	n.d	12.81 ± 2.10
25	2,3-Butanedione	n.d	515.88 ± 36.46	n.d	n.d
26	3-Methyl-2-butanone	663.38 ± 22.46	n.d	n.d	n.d
	Aldehydes				
27	Pentanal	n.d	n.d	n.d	4.70 ± 4.07
28	3-methyl-Butanal	n.d	n.d	1.94 ± 3.36	n.d
29	Heptanal	n.d	n.d	5.41 ± 5.10	60.50 ± 21.06
30	Benzeneacetaldehyde	n.d	n.d	17.72 ± 0.96	20.20 ± 1.70
31	Nonanal	n.d	95.29 ± 6.24	42.02 ± 10.46	135.52 ± 12.14
32	Hexanal	n.d	229.28 ± 26.84	n.d	1033.37 ± 271.55
33	(E)-2-Heptenal	n.d	n.d	n.d	6.32 ± 6.94
	Hydrocarbons				
34	2-methyl-2-nitro-Propane	n.d	825.22 ± 68.42	n.d	n.d
35	propyl-Cyclopropane	n.d	21.48 ± 18.99	n.d	n.d
36	Heptane	n.d	n.d	93.07 ± 33.17	104.00 ± 29.70
37	Tetradecane	n.d	n.d	n.d	13.70 ± 1.88
38	Octane	n.d	n.d	71.31 ± 11.32	68.56 ± 10.43
39	Oxirane, 2-methyl-3-propyl-, trans-	n.d	n.d	231.91 ± 43.37	n.d
40	beta.-Myrcene	n.d	n.d	33.71 ± 14.27	n.d
41	D-Limonene	n.d	n.d	44.51 ± 26.76	15.16 ± 0.92
42	Undecane	n.d	n.d	2.94 ± 2.66	6.67 ± 1.57
43	Tridecane	n.d	n.d	3.84 ± 3.34	7.78 ± 1.39
44	1-Methyl-1,2-epoxycyclohexane	n.d	n.d	n.d	24.53 ± 32.58
45	1-chloro-Pentane	n.d	n.d	n.d	29.82 ± 29.06
46	Dodecane	n.d	n.d	n.d	20.42 ± 3.97
	Other				
47	Toluene	22.34 ± 38.70	57.77 ± 9.28	n.d	n.d
48	Propanoic acid, anhydride	n.d	125.77 ± 217.83	n.d	n.d
49	Oxime-, methoxy-phenyl-	85.75 ± 12.32	90.19 ± 18.42	n.d	n.d

“n.d” means not detected.

Aldehydes were detected higher in both experimental groups than in the control group; aldehydes are mainly produced by fat oxidation and have a low threshold, which plays a key role in sausage flavor formation (29). After 30 days of fermentation, the aldehyde content in the SXS group reached 1260.60 μg/kg, which was significantly higher than that in the CTS group (67.09 μg/kg). In the sausage model, the aldehydes level of the SX group was 324.57 μg/kg, but the CT group did not exhibit any aldehydes. The detection of hexanal and nonanal in the SXS and SX groups suggested that the addition of *Staphylococcus xylosus* might enhance the synthesis of aldehydes. Nonanal has a lemony and citrus flavor, with a content of 95.29 μg/kg in SX and 135.52 μg/kg in SXS. Hexanal produces a grassy and buttery flavor at low levels; however, an undesirable rancid flavor is formed at too high a level (30), with a content of 229.82 μg/kg in SX and 1033.37 μg/kg in SXS, respectively.

Important substances called esters contribute to the creation of sausage flavor. They are produced when alcohols and short-chain fatty acids undergo esterification processes. They also have a low threshold for adding a fruity smell to the product (31). Three different esters were investigated in the SX group. The products might smell sweet and fruity because they include ethyl decanoate, which was detected at 220.96 μg/kg in the SX group, much higher than in the CT group. In the model and sausage ester content change trend is consistent, the experimental group are higher than the control group. This is consistent with the findings of Hu et al. (24), wherein *Lactobacillus* spp. and *Staphylococcus xylosus* were injected into dried sausages from Harbin.

The ketones detected in this experiment were mainly 3-hydroxy-2-butanone, 2-heptanone, 2,3-butanedione, and 3-methyl-2-butanone. 3-Hydroxy-2-butanone is produced by *Lactobacillus* or *Staphylococcus* bacteria through the metabolism of carbohydrate catabolism, and it can impart a fruity flavor to sausages (32). The content of 3-hydroxy-2-butanone substance in SX was 7920.04 μg/kg, which was about 1.5 times higher than that in CT. The same phenomenon was observed in SXS and CTS. The ketone substance threshold is relatively high and plays a role in modifying the flavor of fermented meat products; the results of both *in vivo* and *ex vivo* experiments indicated that the addition of *Staphylococcus xylosus* could enhance the ketone substance content.

Both alcohols and acids play a crucial role in fermented meat products. The former mainly result from the oxidation and degradation of lipid molecules, whereas for components other than unsaturated fatty alcohols, the perception threshold is high and their contribution to the flavor of fermented meat products is weak. The latter originates mainly from the oxidative degradation of glycerol and phospholipids and contributes less to the flavor of fermented meat products. 1-octen-3-ol, with its mushroom aroma and low threshold, is the main alcohol contributing to the flavor of sausages (33), and was detected in both experimental and control groups of sausages. Short-chain fatty acids are crucial for the flavor of sausages, and small levels of acids—mostly isovaleric and acetic acid—were also found in the sausages (34). Large levels of alkanes were also found in the model and following the fermentation of the sausage; however, the alkane threshold was high and did not significantly affect the flavor of the sausage, but it might have a slight effect on the overall flavor.

### 3.8 Analysis of characteristic volatile compounds

**Figure 7:**
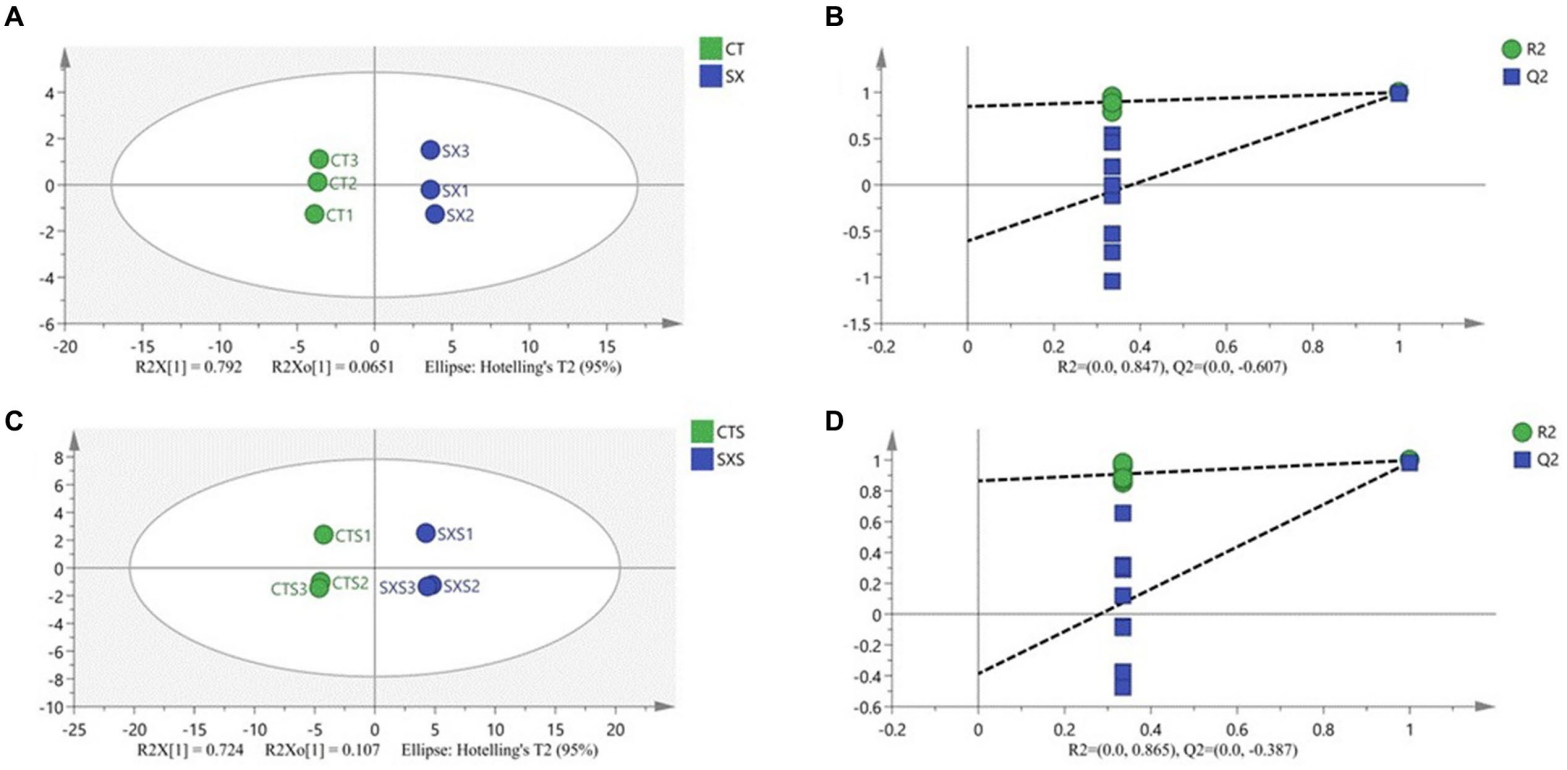
**(A)** Plot of OPLS-DA scores for volatile compounds of the sausage model; **(B)** 200 permutation test of the OPLS-DA model for volatile compounds of the sausage model; **(C)** Plot of OPLS-DA scores for volatile compounds of the sausage; **(D)** 200 permutation test of the OPLS-DA model for volatile compounds of the sausage.

In order to discern distinctive volatile chemicals and provide more insight into the distinctions between the experimental and control groups, a supervised orthogonal partial least squares discriminant analysis (OPLS-DA) model was created. OPLS-DA analysis may effectively differentiate between different treatment groups when volatile flavor compounds are the dependent variable and different treatment groups are the independent variables. The OPLS-DA score graphs for the bratwurst and sausage models are shown in Figure 7, respectively. The sausage model group and the independent variable in this analysis had fit indices (R^2^X) of 0.857 and 0.831, respectively; the dependent variable had fit indices (R^2^y) of 0.999 and 0.998, and the model prediction indices (Q^2^) of 0.991 and 0.986, respectively. Both R^2^ and Q^2^ were greater than 0.5, indicating that the results of the model fit were acceptable. The intersection of the Q^2^ regression line with the vertical axis is less than 0 after 200 permutation tests, as seen in Figures 7B,D. This suggests that the model is not overfitted and that the model validation is successful (35), and it is thought that the results can be applied to the modeling of the sausage as well as the identification and analysis of its volatile flavor compounds.

From the sausage model group, fifteen distinct flavor components were screened at VIP > 1, *p* < 0.05. These included six alcohols (phenylethanol, isopropanol, n-hexanol, isobutanol, 2,3-butanediol, (2R,3R)-(−)-2,3-butanediol), four esters (ethyl caprylate, β-butyrolactone, allylpropyl ester, vinyl acetate), two ketones (3-methyl-2-butanedione, 2,3-butanedione), two aldehydes (nonanal, hexanal), and one hydrocarbon (2-methyl-2-nitropropane).

Twenty distinct flavor components were extracted from the sausages at a significance level of VIP > 1, *p* < 0.05. These included seven alcohols (phenylethanol, n-hexanol, 1-methoxy-5-hexanol, 1,2,5,6-dianhydrogalactitol, (2R,3R)-(−)-2,3-butanediol, 1-octen-3-ol, and linalool), three esters (ethyl hexanoate, ethyl 3-methylbutanoate, formic acid hash), three aldehydes (nonanal, hexanal, heptanal), two ketones (3-hydroxy-2-butanone, 2-heptanone), one acid (acetic acid), and four hydrocarbons (tetradecane, rel-2α*-propyl-3β*-methyloxirane, n-dodecane, laurylene). There were fewer types of described chemicals found in the sausage model’s material environment than in the sausage group because it was more homogeneous than the sausage group. Nonetheless, the sausage model might more accurately represent the variety of sausage taste compounds in terms of the kinds of distinctive flavor compounds assessed.

## 4 Conclusion

By adding *Staphylococcus xylosus* to the sausage model and sausage, we methodically examined the *in vitro* simulation impacts of the sausage model in this study. We also looked at the physicochemical, microbiological, and volatile component features of the sausage model and sausage. The outcomes demonstrated how well the sausage model could replicate the dynamic variations in TBARS, pH, water activity, surface hydrophobicity, and total sulfhydryl concentration found in sausages. When *Staphylococcus xylosus* was added, the pH of the sausage model and sausage decreased, but there was no discernible change in water activity. Both the sausage model and the sausage that had *Staphylococcus xylosus* added had TBARS contents that were noticeably lower than those of the control. The oxidative hydrolysis of proteins during sausage fermentation might be simulated using the sausage model, according to analyses of surface hydrophobicity and total sulfhydryl concentration. The results of the *ex vivo* and *in vivo* experiments indicated that the addition of *Staphylococcus xylosus* could promote the production of 3-hydroxy-2-butanone, while the analysis of the four groups of volatile flavor substances revealed that the changes of ketones and aldehydes in the sausage model and sausage were consistent. The sausage model can accurately replicate the changes in characteristic taste compounds in sausage, according to analysis conducted using the OPLS-DA model for screening characteristic flavors. The *in vivo* reaction through the sausage verified that the strain had the same effect as the *in vitro* reaction, which predicted that the addition of *Staphylococcus xylosus* could accelerate protein hydrolysis, thus promoting the formation of sausage flavor and at the same time delaying fat oxidation, which is beneficial to the quality of the product. The present study demonstrated that the sausage model has a better *in vitro* simulation of the physicochemical properties of sausage; this provides a new research idea for the subsequent investigation of the roles played by microorganisms, endogenous enzymes and bacteriostatic antioxidants in fermented meat products.

## Data availability statement

The original contributions presented in the study are included in the article/supplementary material, further inquiries can be directed to the corresponding author.

## Author contributions

LJ: Conceptualization, Writing – original draft, Writing – review & editing, Data curation. CZ: Data curation, Validation, Writing – original draft, Writing – review & editing. YaZ: Investigation, Visualization, Writing – review & editing. QN: Project administration, Validation, Writing – review & editing. YL: Project administration, Validation, Writing – review & editing. RY: Formal analysis, Project administration, Writing – review & editing. SW: Formal Analysis, Project administration, Writing – review & editing. JN: Supervision, Writing – review & editing. JZ: Investigation, Writing – review & editing. YiZ: Resources, Writing – review & editing.
